# The validity and reliability of the My Jump 2 app for measuring vertical stiffness in male college players

**DOI:** 10.3389/fspor.2024.1405118

**Published:** 2024-05-30

**Authors:** Yizhang Wang, Xintang Wang, Chenglin Luan, Wei Shan, Lijing Gong

**Affiliations:** ^1^ China Institute of Sport and Health Science, Beijing Sport University, Beijing, China; ^2^ Key Laboratory of Exercise and Physical Fitness, Ministry of Education, Beijing Sport University, Beijing, China

**Keywords:** vertical stiffness, drop jump, college players, validity, reliability

## Abstract

**Background:**

Vertical stiffness (K_vert_) can be used to evaluate sports performance and injury risk in players. The My Jump 2 smartphone application (App), is increasingly being used by researchers, coaches, and players in the competitive sports field. We aimed to analyze the reliability and concurrent validity of the My Jump 2 app for measuring K_vert_ in male college players.

**Methods:**

Twenty male college players (10 soccer players, 10 basketball players; age, 20.2 ± 1.3 years old; weight, 76.4 ± 6.0 kg; height, 178.3 ± 4.7 cm) volunteered to take part in this study. Three drop jumps were performed by participants from 30 cm to 40 cm on a force platform and retested after three days. All the jumps were recorded by both the Force platform and the My Jump 2 app. Data obtained from the above two devices were compared using the paired *t* tests, intraclass correlation coefficient (ICC), coefficient of variation (CV), Pearson product moment correlation coefficient (*r*), Bland-Altman plots, and one-way regression.

**Results:**

There was almost perfect agreement between measurement instruments for the K_vert_ value (ICC > 0.972, 95% CI = 0.954–0.992, *P* < 0.01). Almost perfect agreement was observed between evaluators (ICC > 0.989, 95% CI = 0.981–0.997, *P* < 0.05). Also, the My Jump 2 app showed excellent intra-rater reliability in all participants (ICC = 1.000, 95% CI = 1.000–1.000, *P *< 0.001). The My Jump 2 showed good variability when measuring K_vert_ at T1 30 cm (CV = 5.4%), T1 40 cm (CV = 6.7%), T2 30 cm (CV = 5.0%), and T2 40 cm (CV = 10.3%). The test-retest reliability of My Jump 2 was moderate to good at 30 cm (ICC = 0.708, 95% CI = 0.509–0.827); however, it was lower to moderate at 40 cm (ICC = 0.445, 95% CI = 0.222–0.625). Very large correlations were observed between the force platform and the My Jump 2 for K_vert_ (*r* > 0.9655, *P* < 0.001).

**Conclusion:**

The My Jump 2 smartphone application showed excellent reliability and intra-rater consistency in measuring K_vert_ in male college players. While demonstrating excellent intra-rater consistency and strong agreement with force platform measurements, it showed slightly lower reliability at higher jump heights. Overall, the My Jump 2 app is a valid tool for evaluating K_vert_ in college players with careful consideration of its limitations, particularly at higher jump heights.

## 1 Introduction

The concept of stiffness is derived from Hooke's law, which can be described as the ability of objects to resist deformation under external forces (1, 2). Higher stiffness results in lower strain, while lower stiffness results in higher strain. For an organism, increasing stiffness improves the intensity of the supporting tissues, leading to resistance to the forces exerted on the organism. For the human body, stiffness depends on the interactions of muscles, tendons, ligaments, cartilage, bones (3). Vertical stiffness (K_vert_) is a common form to describe lower extremity stiffness. It refers to the degree of deformation of the body's center of gravity under the action of a vertical ground reaction force (vGRF) (4). Athletes who exhibit greater K_vert_ possess greater ability to rapidly extend and retract their lower limbs, which will store more elastic potential energy during the ground contact phase and produce more concentric force output upon leaving the ground (5). K_vert_ has been associated with improved athletic performance in jump contact time, maximum sprint speed, jump height, and endurance running (6–9). K_vert_ is also the strongest predictor of change-of-direction (COD) ability, as recreationally active men with high ability have greater K_vert_ (10).

While optimal stiffness may be necessary for performance, injury may result from either too high or too low stiffness (1). Higher stiffness leads to an increased risk of injury, which may be due to increased impulse and peak forces during exercise as well as reduced joint movement in the lower limbs (11). Similarly, athletes exhibiting poorer lower limb stiffness may increase the potential for soft tissue injuries due to excessive joint motion (9). In addition, Watsford et al. (12) recommended that the assessment of stiffness is an important component of player screening in preparation for professional Australian Rules football.

Soccer is a highly complex sport with tactical, psychological, and physical demands, among which muscle power and strength are highlighted as fundamental factors. Soccer players are frequently required to sprint and change directions on the field. It is estimated that a discontinuous change of motion occurs on average once every 5–6 s during a football match and occurs about 1,000–1,500 times throughout the entire match (13). In a previous study of the Premier League, Drust et al. (14) discovered an average of 19 sprints every 4–5 min during a match, and Bloomfield et al. (15) observed that 727 (±203) CODs were required for an entire match, with an average of one occurring every 8 s. Basketball is also a court-based team sport that is highly intermittent, with players changing movements approximately every 1–3 s (16–18). High intermittent requirements in basketball involve frequent transitions between low intensity activities and intense bursts of sprinting. Specifically, high-intensity movements such as intense shuffling, sprinting, and jumping comprise 8.8%, 5.3%, and 2.1% of total playing time, respectively (19). Simultaneously, COD tasks constitute 20.7% of sprinting activity in basketball (20).

Force platforms remain the gold standard for vGRF, time of contact, and time of flight determination, from which K_vert_ can be calculated (21, 22). However, force platforms are expensive, inconvenient to transport, limited in use to laboratory environments, and require testers to have solid professional knowledge when performing tests and analyzing data (23), which may become a barrier between research and practice. Thus, there is an important demand for accessible, easy-to-use, and low-cost equipment to evaluate K_vert_. My Jump 2 is a mobile application for iOS devices utilizing the video camera system for the assessment of K_vert_ (24). The app is affordable, practical, and can be used in different fields (23, 25). This tool already showed high reliability and reproducibility for evaluating the jump height in drop jumps (DJs), countermovement jumps (CMJs), and squat jumps (SJs) compared with force platforms and high-speed video cameras in recreationally active subjects, athletes, and primary school children (26–28). However, no information about the validity and reliability of the K_vert_ is available.

Therefore, the aim of the study was to analyze the validity and reliability of the My Jump 2 app for measuring the K_vert_ in male college players. It was hypothesized that the My Jump 2 app would have good reliability and concurrent validity compared to the Force platform for measuring the K_vert_ in male college players.

## 2 Methods

### 2.1 Participants

Twenty male college players (10 soccer players, 10 basketball players; age, 20.2 ± 1.3years; weight, 76.4 ± 6.0 kg, height, 178.3 ± 4.7 cm) were recruited for this study. All participants had at least 3 years of football or basketball training experience and were skilled in the drop jump. Maintain a training frequency of 3 times a week or more in the last 3 months without any serious lower limb injuries (tendon, ligament, knee, ankle, etc. injuries resulting in an inability to engage in regular physical activity for the past 3 months). The total sample size required was calculated to be 12 using G*Power 3.1.9.2 software (Es = 0.5, *α* = 0.05, Power = 0.80). There are several possible reasons for the potential attrition: First, some subjects may not be as compliant as they should be; second, there may be a loss of sample size due to injuries to subjects during the experiment. Considering the attrition rate of the sample size, a total of 20 participants were included. They were healthy, with no cardiac or pulmonary problems and no injuries on the day of the test. They had no strength, jumping, or high-intensity training for 48 h prior to the assessment. Before the test began, they signed a written informed consent form after being briefed about it and made comfortable with its processes.

The present study was carried out in accordance with human experimentation guidelines (Helsinki Declaration); it received no specific grant from funding agencies in the public, commercial, or not-for-profit sectors and was approved by the Beijing Sport University ethics committee (2023161H). The authors have no relationship with the app or its creators.

### 2.2 Procedures

The participants carried out a standardized 10-min warm-up prior to testing (Table 1). The first step is to use the cycle ergometer for body heat generation, followed by dynamic stretching and core activation, and finally a number of sets of specialized warm-ups. Their body mass was measured to the nearest 0.1 kg with a force platform. Then, each participant performed three DJs at two heights (30 cm and 40 cm) onto a force platform (Kistler 9287 BA, Kistler Instruments Ltd., Hook, UK) whilst simultaneously being recorded with a smartphone using the My Jump 2 app (The First Day of Testing, T1). DJ was performed from the top of a 30/40 cm box placed 10 cm from the edge of the force platform, with the 30 cm DJ followed by the 40 cm DJ. The participants were asked to fall down a step with no initial velocity, land, and jump upwards as fast and as high as possible, keeping both hands on the hips throughout the fall and jump. Two minutes of passive rest between attempts (24). Shoes and socks have a significant effect on K_vert_, so this experiment required participants to remove their shoes and socks before the test (29). After 3 days, they were asked to repeat the same test procedure as on day one (The Second Day of Testing, T2).

**Table 1:** Standardized warm-up protocol.

Exercise	Sets * repetitions
Cycle ergometer	5 min
Lunge twist	2*5(each side)
Lateral lunge	2*5(each side)
Inchworms	10
Free squat	2*10
Drop lands	1*4
Drop jumps	1*4 (75% maximal perceived effort)

### 2.3 My Jump 2 App

**Figure 1:**
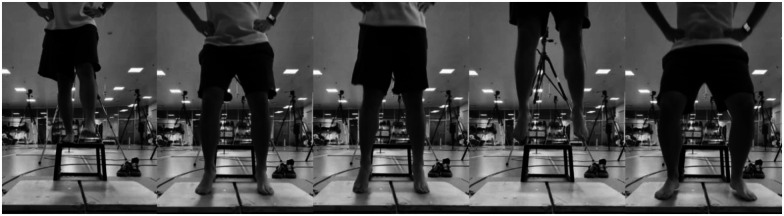
Drop jump test.

The app for the iOS operating system (Apple Inc., Cupertino, CA) was developed using the software (XCode0.5 for Mac OS X 10.9.2; Apple Inc.) and installed on the iPhone 13 Pro (Apple Inc.). The evaluation required a high-speed (240 Hz) and 1080-pixel-quality camera that comes with the smartphone. The app analyzed the K_vert_ by computing the time among 3 frames (in milliseconds) manually selected by the evaluator, which were the initial contact frame, the take-off frame from the floor, and the final landing frame through the video (Figure 1). A number of data points were recorded from the app, including jump height (cm), contact time (T_c_, ms), flight time (T_f_, ms), and K_vert_ (kN/m).

All collections were made with the same smartphone, and the captured video was analyzed independently by two evaluators with no professional experience in video analysis. The evaluator was always recording from the same position and with the same distance from the participants (1.5 m) as standard calibration according to the manufactory instructions (26). The smartphone is placed on a tripod.

### 2.4 Force platform

A 400 × 600 mm force platform (Kistler 9287 BA, Kistler Instruments Ltd., Hook, UK) measures the vGRF and contact time of the DJ at a sampling frequency of 1,000 Hz. The force platform was connected to a portable computer equipped with the software to analyze the force data (BioWare V5.3.2.9, Kistler Holding AG, Switzerland). The maximum force (F_max_) generated during the athlete's DJ is manually selected from the data recorded by the force platform. The first time when vGRF exceeds 10 N is selected as the time when the foot touches the ground, and the first time when vGRF is lower than 10 N indicates the time when the foot leaves the force platform, thus the contact time is derived. F_max_ and contact times from the analysis of the force platform data were used to calculate K_vert_ using the equation:

$$K_{\mathrm{vert}}=\frac{F_{\max}}{\Delta y}$$

$$\Delta y=\frac{\left(F_{\max}*T_{c}^{2}\right)}{\left(m*\pi^{2}\right)}-\frac{g*T_{c}^{2}}{8}$$

where K_vert_ stands for K_vert_ (kN/m), F_max_ stands for maximum force (*N*), Δ*y* stands for vertical displacement of the center of gravity (m), and T_c_ stands for the contact time (ms).

### 2.5 Statistical analysis

Descriptive statistics were presented using means and standard deviations. A Shapiro-Wilk test was used to check the data's normality. The intraclass correlation coefficient (ICC) was conducted to assess the reproducibility of My Jump 2 app for measuring vertical stiffness. Therefore, the inter-rater, intra-rater, and test-retest reliability were tested by ICC (2,1). Bland-Altman plots (30) were created to show the agreement between the two testing methods. To measure the variability of My Jump 2 for all jumps performed at both heights, the coefficient of variation (CV) was used. The formula for the effect size was Cohen' *d* = (Mean _group1_—Mean _group2_)/SD _(combined group or group1)_, which was evaluated on the basis of the criteria of 0.2–0.49 as small, 0.5–0.79 as medium, and >0.8 as large (30). To test the concurrent validity of My Jump 2, Pearson's product-moment correlation coefficient (*r*) was performed on normally distributed data. The paired *t* tests were applied to compare the mean differences (MD) in K_vert_ between the two devices. MD < 0 indicates that the experimental equipment underestimates the true value, and MD > 0 indicates that the experimental equipment overestimates the true value. The presence of proportional error between the two devices was analyzed using one-way linear regression, and *r*^2^ > 0.1 was judged significant (31). If a proportional error exists between the two devices, it indicates that the error between the two devices is inconsistent when measured at the depth of jump height specified in this experiment. The statistical significance was fixed at the *P *≤ 0.05 level. All calculations were performed using IBM SPSS Statistics 25 (IBM Co., USA) and GraphPad Prism 8.3.0 for Windows.

## 3 Results

All jump tests were successfully completed by participants. A total of 240 jumps were included in the next analysis. During testing, no adverse events were recorded. All data are normally distributed from the results of the P-P plot and the Shapiro-Wilk test (*P* > 0.05).

### 3.1 Reliability

#### 3.1.1 Inter-rater reliability

There was almost perfect agreement between two evaluators of My Jump 2 app at each category, which showed significant differences with small effect sizes (T1 30 cm: ICC = 0.994 (95% CI: 0.990–0.997, *P* < 0.05, ES = 0.03);T2 30 cm: ICC = 0.992 (95% CI: 0.985–0.995, *P* < 0.05, ES = 0.04);T1 40 cm: ICC = 0.989 (95% CI: 0.981–0.994, *P* < 0.05, ES = 0.05);T2 40 cm: ICC = 0.995 (95% CI: 0.991–0.997, *P* < 0.05, ES = 0.03) (Table 2).

**Table 2:** Stiffness data and inter-rater reliability of the My Jump 2.

Variable	Evaluator A(kN/m)	Evaluator B(kN/m)	ICC (95% CI)	MD (95% CI, cm)
T1 30 cm	19.35 ± 2.53	19.26 ± 2.54	0.994 (0.990–0.997)	−0.081 (−0.148 to −0.015)
T2 30 cm	20.22 ± 2.84	20.11 ± 2.75	0.992 (0.985–0.995)	−0.106 (−0.197 to 0.016)
T1 40 cm	19.57 ± 2.47	19.46 ± 2.45	0.989 (0.981–0.994)	−0.113 (−0.202 to −0.023)
T2 40 cm	20.23 ± 3.31	20.13 ± 3.27	0.995 (0.991–0.997)	−0.101 (−0.184 to −0.018)

T1 30 cm, the first test in 30 cm; T2 30 cm, the second test in 30 cm; T1 40 cm, the first test in 40 cm; T2 40 cm, the second test in 40 cm; ICC, intraclass correlation coefficient; MD, mean difference.

#### 3.1.2 Intra-rater reliability

The My Jump 2 app showed excellent intra-rater reliability in all participants [T1 30 cm, T2 30 cm, T1 40 cm,T1 40 cm: ICC = 1.000 (95% CI: 1.000–1.000, *P* > 0.05)] (Table 3).

**Table 3:** Stiffness data and intra-rater reliability of the My Jump 2.

Variable	FTA (kN/m)	STA (kN/m)	ICC (95% CI)	MD (95% CI, cm)
T1 30 cm	19.35 ± 2.53	19.35 ± 2.53	1.000 (1.000–1.000)	−0.002 (−0.001 to 0.005)
T2 30 cm	19.57 ± 2.47	19.58 ± 2.47	1.000 (1.000–1.000)	0.003 (−0.001 to 0.006)
T1 40 cm	20.22 ± 2.84	20.22 ± 2.84	1.000 (1.000–1.000)	0.004 (0.0004–0.007)
T2 40 cm	20.23 ± 3.31	20.23 ± 3.31	1.000 (1.000–1.000)	0.004 (0.001–0.006)

T1 30 cm, the first test in 30 cm; T2 30 cm, the second test in 30 cm; T1 40 cm, the first test in 40 cm; T2 40 cm, the second test in 40 cm; FTA, first time analysis; STA, second time analysis; ICC, intraclass correlation coefficient; MD, mean difference.

#### 3.1.3 Test-retest reliability and CV

At a drop height of 30 cm, the ICC was 0.708 (95% CI: 0.509–0.827, *P* < 0.01). The two tests demonstrated moderate-to-good agreement. ES = 0.35 indicated a small-to-medium impact magnitude. The CV for the T1 30 cm and T2 30 cm groups were 5.4% and 5.0%, respectively, which were within acceptable ranges. The ICC = 0.445 (95% CI: 0.222–0.625, *P* < 0.01) was observed when the drop height was 40 cm. The two tests showed low to moderate agreement. The effect size, ES = 0.24, showed a small-to-medium effect size. The CV for the T1 40 cm and T2 40 cm groups were 6.7% and 10.3%, respectively, which were close to the threshold of the acceptable range (Table 4).

**Table 4:** Stiffness data and test-retest reliability of the My Jump 2.

Variable	My Jump 2(kN/m)	ICC (95% CI)	CV (%)
T1 30 cm	19.35 ± 2.53	0.708 (0.509–0.827)	5.4
T2 30 cm	20.22 ± 2.84		5.0
T1 40 cm	19.57 ± 2.47	0.445 (0.222–0.625)	6.7
T2 40 cm	20.16 ± 3.17		10.3

T1 30 cm, the first test in 30 cm; T2 30 cm, the second test in 30 cm; T1 40 cm, the first test in 40 cm; T2 40 cm, the second test in 40 cm; ICC, intraclass correlation coefficient; CV, coefficient of variation.

### 3.2 Concurrent validity

**Figure 2:**
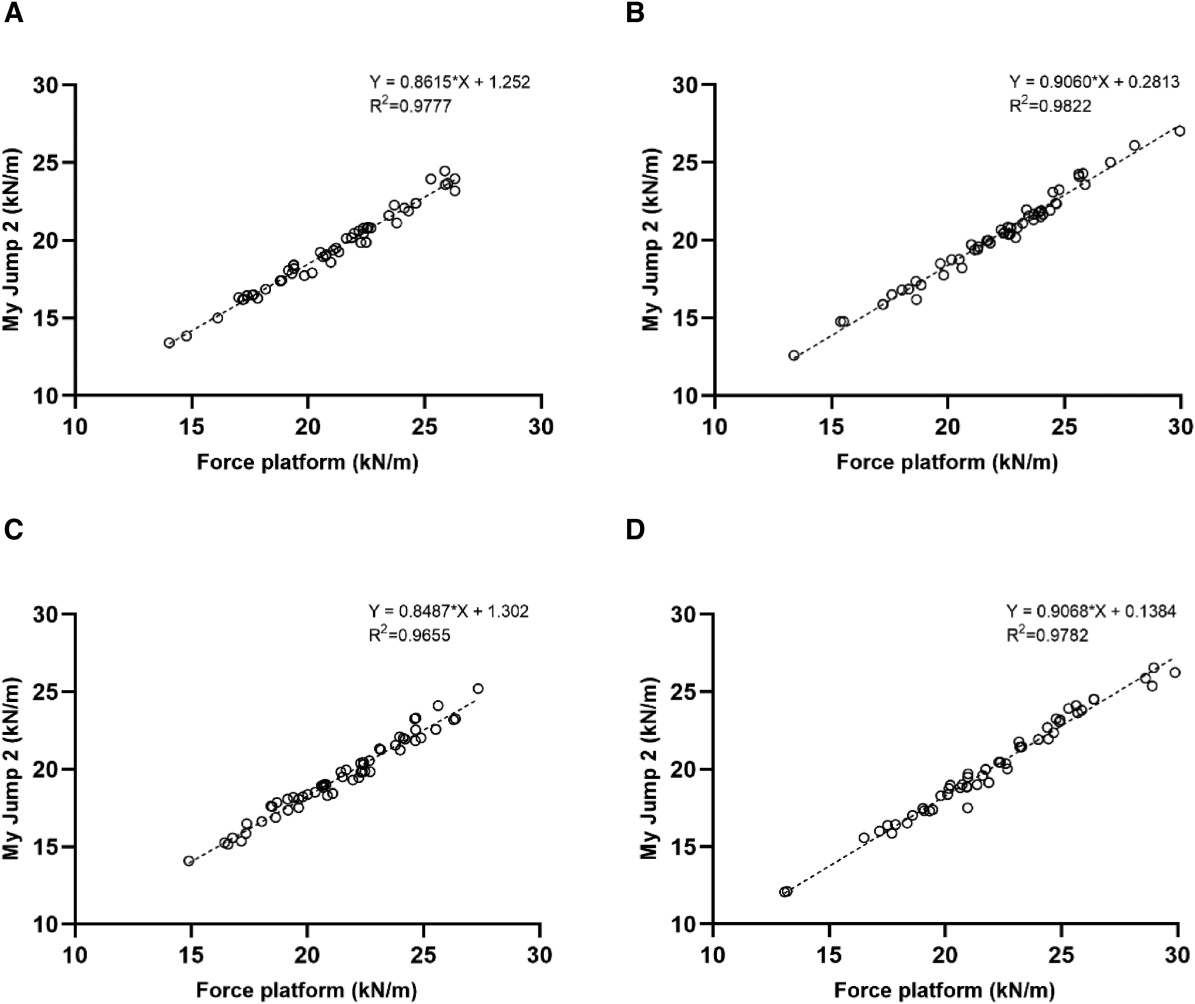
Correlation between My Jump 2 app and the force platform in T1 30 cm (**A**), T2 30 cm (**B**), T1 40 cm (**C**), T2 40 cm (**D**).

There was almost perfect agreement between the My Jump 2 app and the force platform measures of K_vert_ in each group, which revealed highly significant differences with medium effect sizes (T1 30cm: ICC = 0.979 (95% CI: 0.966–0.988, *P* < 0.01, ES = 0.58); T2 30 cm: ICC = 0.987 (95% CI: 0.978–0.992, *P* < 0.01, ES = 0.58); T1 40 cm: 0.972 (95% CI: 0.954–0.983, *P* < 0.01, ES = 0.69); T2 40 cm: 0.985 (95% CI: 0.976–0.991, *P* < 0.01, ES = 0.56) (Table 5). Also, the Pearson product moment correlation coefficient (*r*) showed almost perfect correlation between the My Jump 2 and the force platform measurements for K_vert_ in each group (T1 30 cm: *r* = 0.989, *P* < 0.01; T2 30 cm: *r* = 0.991, *P* < 0.01; T1 40 cm: *r* = 0.983, *P* < 0.01; T2 40 cm: *r* = 0.989, *P* < 0.01) (Figure 2).

**Table 5:** Stiffness data and validaty of the My Jump 2.

Variable	Kistler (kN/m)	My Jump 2 (kN/m)	ICC (95% CI)	MD (95% CI, kN/m)	ES	*r*
T1 30 cm	21.00 ± 2.90	19.35 ± 2.53	0.979 (0.966–0.988)	−1.657 (−1.799 to 1.574)	0.58	0.989
T2 30 cm	22.01 ± 3.10	20.22 ± 2.84	0.987 (0.978–0.992)	−1.788 (−1.911 to 1.664)	0.58	0.991
T1 40 cm	21.53 ± 2.85	19.57 ± 2.74	0.972 (0.954–0.983)	−1.954 (−2.117 to 1.792)	0.69	0.983
T2 40 cm	22.08 ± 3.46	20.16 ± 3.17	0.985 (0.976–0.991)	−1.920 (−2.067 to 1.772)	0.56	0.989

T1 30 cm, the first test in 30 cm; T2 30 cm, the second test in 30 cm; T1 40 cm, the first test in 40 cm; T2 40 cm, the second test in 40 cm; ICC, intraclass correlation coefficient; MD, mean difference; ES, effect size; *r*, Pearson's product moment correlation coefficient.

**Figure 3:**
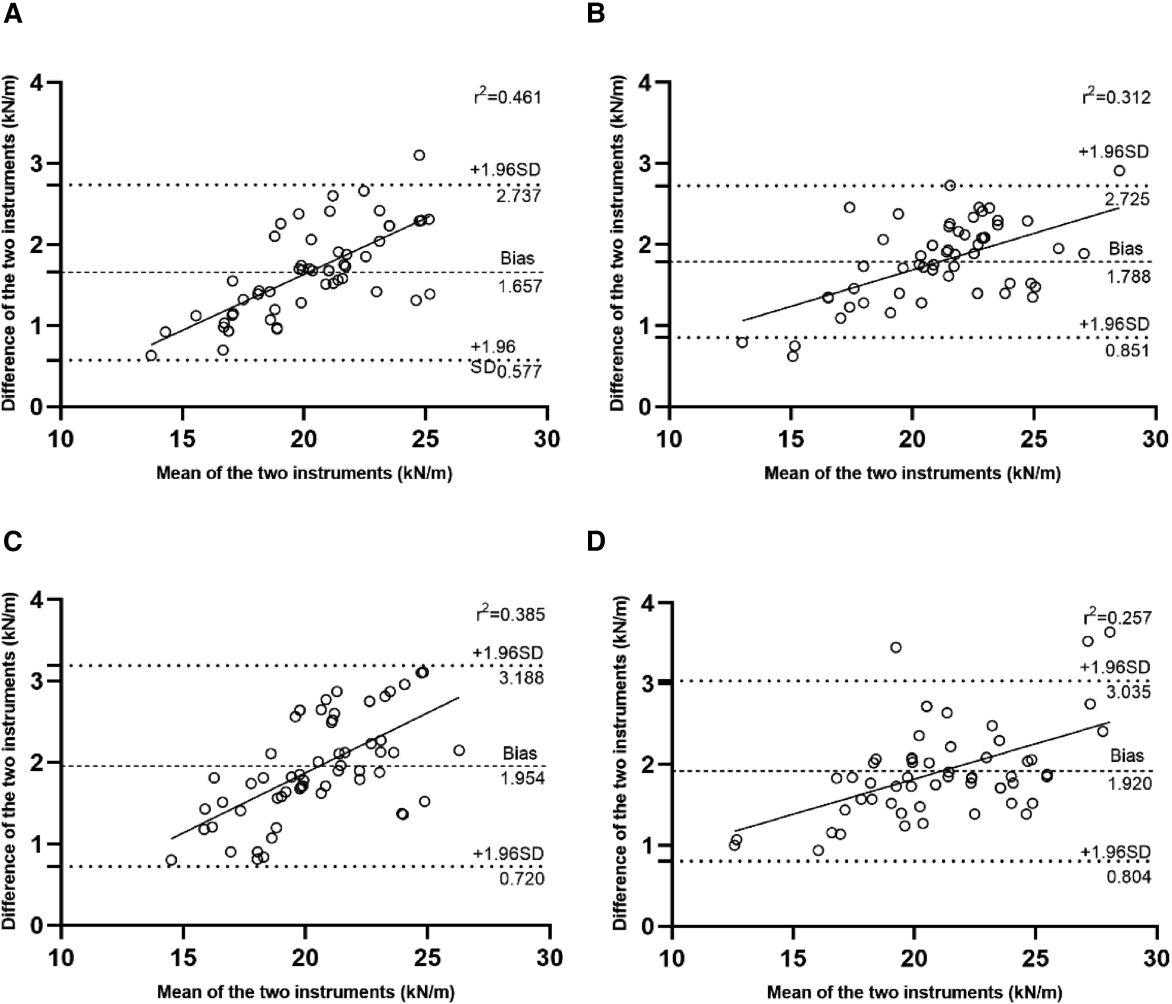
Bland–Altman plots for the force platform and My Jump 2 app (**A**) T1 30 cm, (**B**) T2 30 cm, (**C**) T1 40 cm, (**D**) T2 40 cm. The central line represents the absolute average difference between instruments, while the upper and the lower lines represent ±1.96 standard deviation (SD).

Figure 3 shows the limits of agreement between the My Jump 2 and force platform measures in T1 30 cm, T2 30 cm, T1 40 cm, and T2 40 cm. The charts indicate that 1/60 (1.7%), 4/60 (6.7%), 0/60 (0%), and 3/60 (5%) of the data points were beyond the mean ± 1.96 SD lines in T1 30 cm, T2 30 cm, T1 40 cm, and T2 40 cm, respectively. The results of the regression analysis in the Bland-Altman plots reveals that there is a proportional error in the K_vert_ measured by the My Jump 2 APP at different jump depth heights. The determination coefficients for each group are *r*^2^ = 0.461 (T1 30 cm), *r*^2 ^= 0.312 (T2 30 cm), *r*^2^ = 0.385 (T1 40 cm), and *r*^2^ = 0.257 (T2 40 cm), all >0.1, indicating that the error between the two devices is inconsistent when measured at the depth of jump height specified in this experiment. The larger the measured K_vert_ value, the greater the error between the two devices.

**Figure 4:**
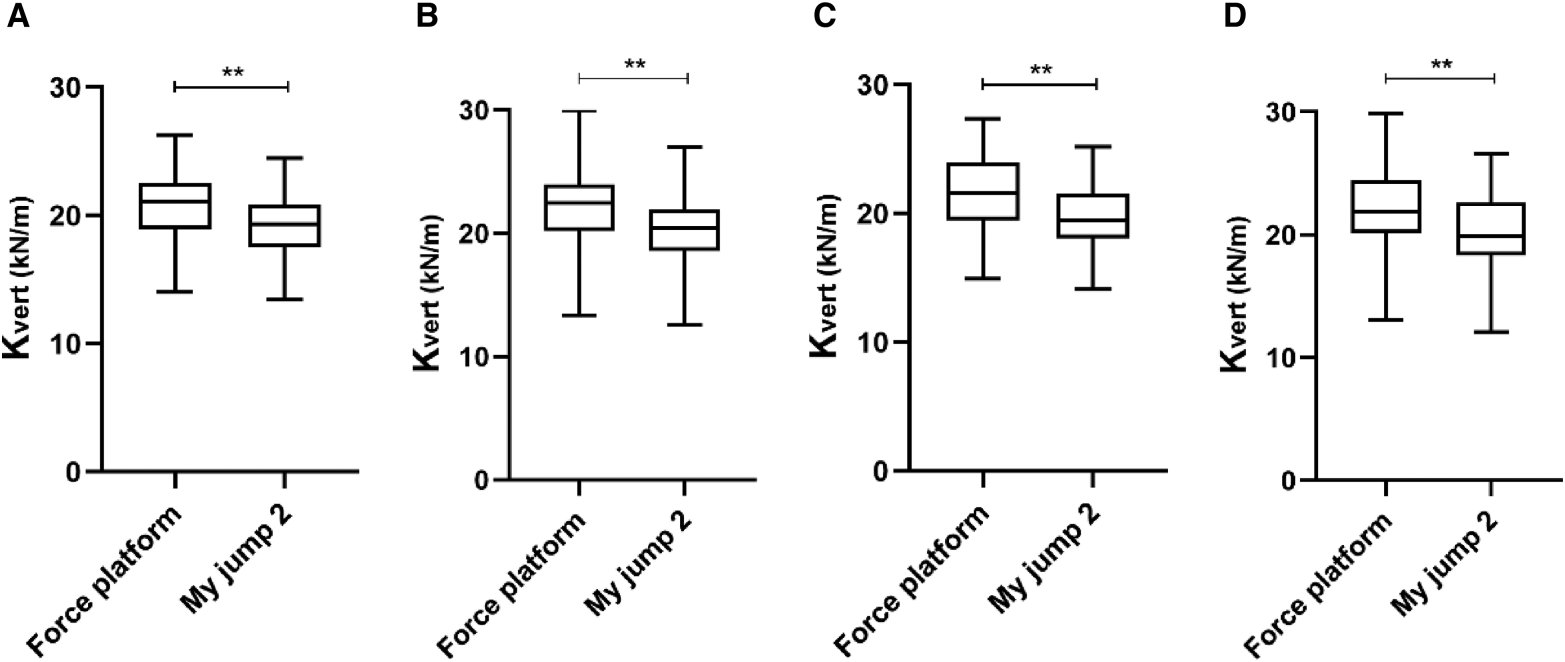
Comparison between My Jump 2 app and the force platform in T1 30 cm (**A**), T2 30 cm (**B**), T1 40 cm (**C**), T2 40 cm (**D**).

The results of the paired samples *t*-test showed that there were significant differences between the My Jump 2 app and the Force platform in K_vert_ for all of the variables (T1 30 cm, 21.00 ± 2.90 vs. 19.35 ± 2.53 kN/m, MD = −1.657, *P *< 0.01; T1 40, 21.53 ± 2.85 vs. 19.57 ± 2.74 kN/m, MD = −1.954, *P *< 0.01; T2 30, 22.01 ± 3.10 vs. 20.22 ± 2.84 kN/m, MD = −1.788, *P *< 0.01; T2 40, 22.08 ± 3.46 vs. 20.16 ± 3.17 kN/m, MD = −1.920, *P *< 0.01) (Figure 4). It is demonstrated that the K_vert_ recorded by My Jump 2 App is much lower than those reported by force platforms.

### 3.3 Establish regression equation

**Figure 5:**
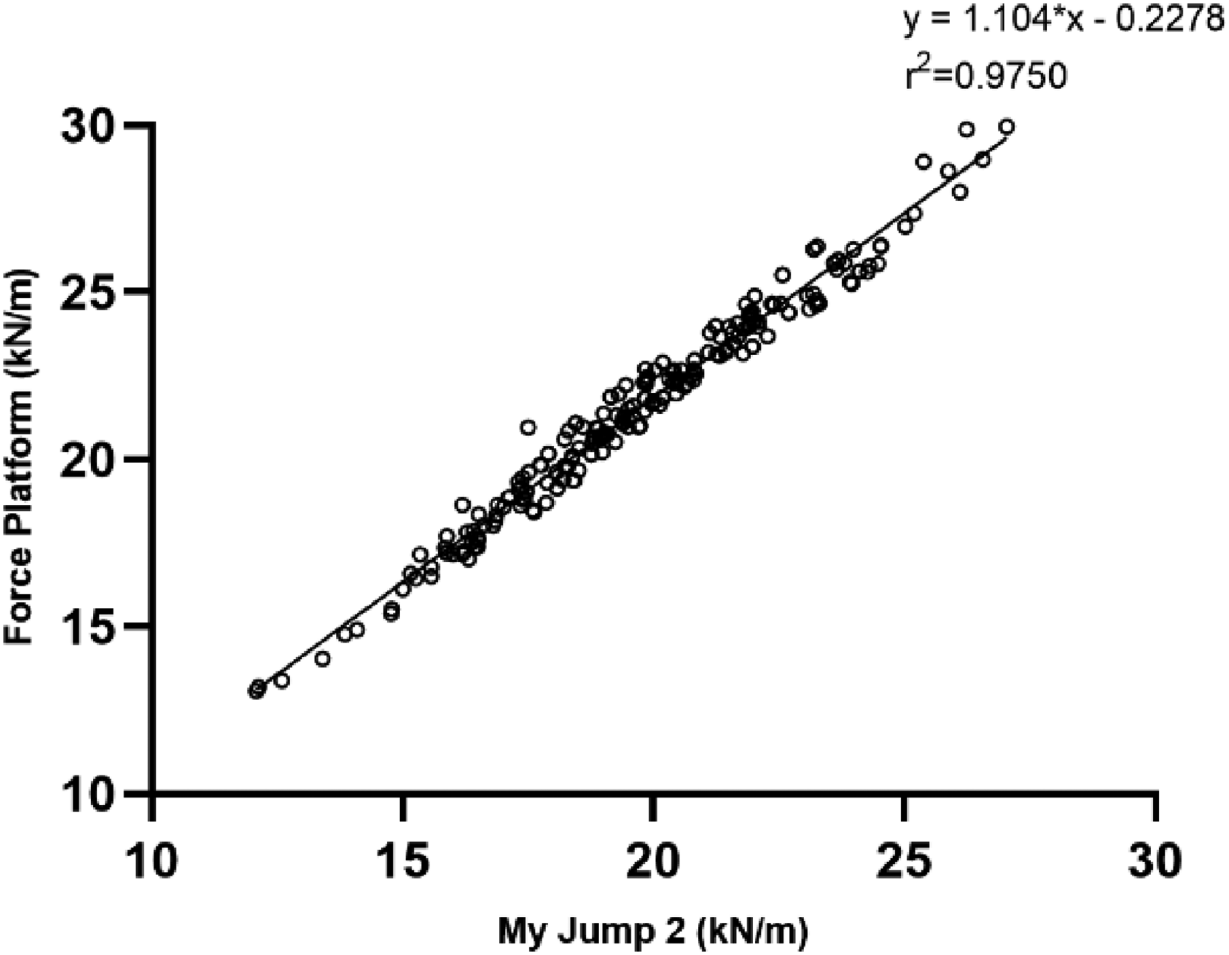
One-way linear regression plot of force platform measurements and My Jump 2 APP measurements.

In order to reduce such systematic errors due to the device, we can make My Jump 2 APP measurements closer to the real values by building regression equations. We conducted a monotonic regression analysis of stiffness data recorded by the force platform on all individuals having stiffness data measured by the My Jump 2 APP (240 each, 480 overall) and derived the following one-way regression equation: *y* = 1.104*x-0.2278 (x: My Jump 2 APP data, y: Kistler 3D force platform data). During later updates of the App, the developer can update the algorithm with this equation in order to provide more accurate measurements to the user (Figure 5).

## 4 Discussion

The purpose of this study was to analyze the concurrent validity and reliability of My Jump 2 for measuring K_vert_ performance in male college soccer and basketball players. The main findings showed that My Jump 2 app has high validity and intra-rater reliability for measuring K_vert_ in male college soccer and basketball players. However, the test-retest reliability for jumps at 40 cm was found to be inconsistent, ranging from poor to moderate. Additionally, there were significant differences between the two devices for measuring K_vert_ from both 30 cm and 40 cm jump heights.

To our knowledge, this is the first study testing the reliability and validity of the K_vert_ performance of the My Jump 2 among male college players. Previous studies had compared the effects of the My Jump 2 to force platform measurement on a number of different jumps; for example, Gallardo-Fuentes et al. (27) found a near-perfect correlation (*r* = 0.97–0.99) in jump height, along with very strong levels of agreement (ICC = 0.98–0.99) and a small mean difference between devices (0.1 ± 0.8 cm) when testing CMJ, SJ, and DJ in both male and female athletes; Haynes et al. (25) examined the RSI of recreational athletes performing DJs, finding a near-perfect correlation (20 cm, *r* = 0.94; 40 cm, *r* = 0.97) and intraclass correlation coefficients (20 cm, ICC = 0.95; 40 cm, ICC = 0.98); Barbalho et al. (32) observed that, compared with the force platform, the My Jump 2 app tested showed excellent reliability for the drop jump's flight time and interlimb asymmetry (ICC > 0.98). For interlimb contact-time asymmetry, the values were 18.4 (9.9) and 19.1 (9.9) milliseconds for the My Jump 2 app and the force platform, respectively (*P* = 0.88). For flight-time asymmetries, the values were 389.7 (114.3) and 396.8 (112.5) milliseconds for the My Jump 2 app and the force platform, respectively (*P* = 0.88). All of the above studies suggest that the My Jump 2 is able to reliably measure DJ performance in a wide range of populations, from elite athletes to more recreational athletes with varied abilities in jumping technique.

The similarity between this study and previous studies is that the object of the study is the my jump 2 app, but the difference is that the metrics studied are different (24–26, 32). This study focuses on the reliability and validity of the app for vertical stiffness testing. Vertical stiffness was chosen for this study because it has been linked to both sports performance and injuries (5–10). Compared to RSI, CMJ and other commonly studied metrics, there is a lack of research on the role of vertical stiffness in sports.

When the drop height was 30 cm, the two tests before and after showed moderate to good agreement. Although significant differences were observed, the effect sizes were small, and the coefficients of variation were within acceptable ranges, which indicated that the retest reliability and stability of the My Jump 2 APP were better at the 30 cm test. When the drop height was 40 cm, the two tests before and after indicated low to moderate agreement. At the same time, both sets of coefficients of variation increased, even beyond the threshold of the acceptable range, which might be related to the increase in drop height. The coefficient of variation measures the internal stability of the data, i.e., the degree of dispersion. Due to the fact that the experiment required slippers and socks to be removed and the hardness of the force platform surface, individuals had diverse proprioceptive experiences when falling at different jump depths (29). Participants testing at 40 cm may experience increased instability in the landing phase due to the greater impact of the landing.

It is demonstrated that the K_vert_ recorded by My Jump 2 App is much lower than those reported by force platforms. This result is similar to that of Balsalobre et al. (26) when they used the My jump 2 App for CMJ height measurements. The explanation for this discrepancy may be that the experimental mobile phone supports a maximum frame rate of 240 Hz for slow motion video recording. However, the sampling frequency of the Kistler 3D force platform is 1,000 Hz, and since the sampling frequency of the My Jump 2 APP is lower than that of the Kistler 3D force platform, there will be an error in determining a few key frames through the video recorded by the My Jump 2 APP, leading to a deviation in the final results. There are two ways to reduce this error. Firstly, use a smartphone with a higher frame rate camera. Secondly, the algorithm of the software was updated to reduce the error between the two devices by creating a univariate regression equation (which was used in this experiment).

The force platform is regarded as the “gold standard” test method for measuring jumping performance, but its use is limited by circumstance, economics, and expertise. This study found that the My Jump 2 app is an easy and portable alternative to the force platform for assessing K_vert_. In practice, we should also pay attention to the following issues. The experiment was conducted with the smartphone on a tripod and placed 1.5 m in front of the participant. However, the height of the smartphone above the ground was not specified. In fact, it has been demonstrated that there is no significant difference in the results obtained from measurements taken at different heights above the ground when tested using the My jump2 app (33). Therefore, if a tripod is not available in a realistic measurement environment, it is feasible for the evaluator to conduct the test with a mobile phone in hand.

A possible limitation of our study was that although the latest versions of the phone have been equipped with cameras that can record 240 Hz slow motion, it is still possible that the exact landing and take-off frames could be missed. If the accuracy of the K_vert_ is to be increased again, a higher frame rate camera is required for video capture. However, there are some problems associated with this, for example, the use of a camera with a higher frame rate may entail a greater cost. Therefore, when making the choice of experimental equipment in the future, how can we choose a cost-effective experimental equipment is a matter of concern. This equipment can satisfy the requirement of accuracy and at the same time keep the cost within a certain range. Another limitation was that the vertical displacement of the center of gravity (Δy) in this study was derived from the equation. Although it is reasonable to use this method to calculate the vertical displacement of the center of gravity, this is, after all, a result processed through mathematical methods, and the potential for bias increases (34). Therefore, in subsequent studies, the use of motion capture systems to obtain the maximum vertical displacement of the center of gravity of the body can be considered. A final limitation was that this study only investigated the app's reliability and validity for K_vert_ measurements in soccer and basketball players; in the future, K_vert_ measurements can be performed and validated in a diverse range of athletes from various sports to broaden the application's scope.

## 5 Conclusions

The My Jump 2 smartphone application showed excellent reliability and intra-rater consistency in measuring K_vert_ in male college players. While demonstrating excellent intra-rater consistency and strong agreement with force platform measurements, it showed slightly lower reliability at higher jump heights. Overall, the My Jump 2 app is a valid tool for evaluating K_vert_ in college players with careful consideration of its limitations, particularly at higher jump heights.

## Data Availability

The original contributions presented in the study are included in the article/Supplementary Material, further inquiries can be directed to the corresponding author.
